# Functional Classification of Fusion Proteins in Sarcoma

**DOI:** 10.3390/cancers16071355

**Published:** 2024-03-29

**Authors:** Marco Wachtel, Didier Surdez, Thomas G. P. Grünewald, Beat W. Schäfer

**Affiliations:** 1 Department of Oncology and Children’s Research Center, University Children’s Hospital, Steinwiesstrasse 75, CH-8032 Zurich, Switzerland; 2 Balgrist University Hospital, Faculty of Medicine, University of Zurich (UZH), CH-8008 Zurich, Switzerland; 3 Division of Translational Pediatric Sarcoma Research, German Cancer Research Center (DKFZ), German Cancer Consortium (DKTK), 69120 Heidelberg, Germany; 4 Hopp-Children’s Cancer Center (KiTZ), 69120 Heidelberg, Germany; 5 National Center for Tumor Diseases (NCT), NCT Heidelberg, a Partnership between DKFZ and Heidelberg University Hospital, 69120 Heidelberg, Germany; 6 Institute of Pathology, Heidelberg University Hospital, 69120 Heidelberg, Germany

**Keywords:** sarcoma, fusion proteins, transcription factors, kinases, epigenetic regulators, mesenchymal lineage

## Abstract

**Simple Summary:**

Fusion proteins are an important class of oncogenes in sarcoma. Approximately 40 percent of all sarcoma entities are driven by one of the more than 100 known fusion proteins. Individual fusion proteins belong to different protein classes, including transcription factors, epigenetic regulators and kinases, and are characterized by specific mechanisms of action. Here, we summarize the current knowledge on fusion oncogenes in sarcoma, highlighting the differences as well as similarities in their biology. Overall, this work provides the basis for a functional classification of sarcomas.

**Abstract:**

Sarcomas comprise a heterogeneous group of malignant tumors of mesenchymal origin. More than 80 entities are associated with different mesenchymal lineages. Sarcomas with fibroblastic, muscle, bone, vascular, adipocytic, and other characteristics are distinguished. Nearly half of all entities contain specific chromosomal translocations that give rise to fusion proteins. These are mostly pathognomonic, and their detection by various molecular techniques supports histopathologic classification. Moreover, the fusion proteins act as oncogenic drivers, and their blockade represents a promising therapeutic approach. This review summarizes the current knowledge on fusion proteins in sarcoma. We categorize the different fusion proteins into functional classes, including kinases, epigenetic regulators, and transcription factors, and describe their mechanisms of action. Interestingly, while fusion proteins acting as transcription factors are found in all mesenchymal lineages, the others have a more restricted pattern. Most kinase-driven sarcomas belong to the fibroblastic/myofibroblastic lineage. Fusion proteins with an epigenetic function are mainly associated with sarcomas of unclear differentiation, suggesting that epigenetic dysregulation leads to a major change in cell identity. Comparison of mechanisms of action reveals recurrent functional modes, including antagonism of Polycomb activity by fusion proteins with epigenetic activity and recruitment of histone acetyltransferases by fusion transcription factors of the myogenic lineage. Finally, based on their biology, we describe potential approaches to block the activity of fusion proteins for therapeutic intervention. Overall, our work highlights differences as well as similarities in the biology of fusion proteins from different sarcomas and provides the basis for a functional classification.

## 1. Introduction

**Figure 1:**
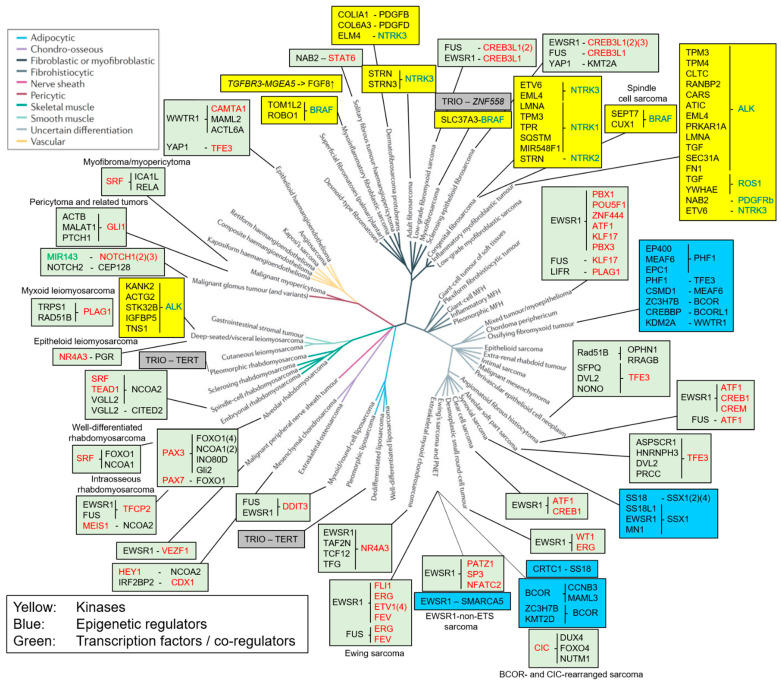
Phylogeny depicting about 60 different sarcoma entities or mesenchymal neoplasms and associated fusion proteins (adapted from [3]). Branch colors indicating the different cell lineages are defined in the top left inset. Yellow boxes represent fusions of kinases/growth factors, with kinase parts depicted in blue, blue boxes represent fusions of epigenetic regulators, green boxes represent fusions of transcription factors or transcriptional co-regulators, with the DNA binding moiety depicted in red, and grey boxes represent fusion proteins acting by an unknown mechanism of action. MicroRNA is depicted in green. Fusions depicted in italics do not produce a functional protein.

Sarcomas represent one out of five general classes of tumors and comprise all cancer entities that are of mesenchymal origin [1]. Mesenchymal tissues include connective tissue (adipose tissue, bone and cartilage), smooth and skeletal muscle, as well as the lymphatic and blood vessels. Together, these tissues contain a great variety of different cell types, including osteocytes, adipocytes, chondrocytes, muscle cells, fibroblasts, neural cells and stromal cells [2]. This cellular diversity is reflected by the heterogeneity of sarcomas. Based on histological and/or molecular characteristics, many of the different sarcoma entities can be associated with one of these normal mesenchymal cell types, suggesting that the different sarcoma entities represent the malignant counterparts of the related normal cells (Figure 1) [3].

In adults, sarcomas account for about one percent of all malignant neoplasms, while in children it is nearly 20 percent [4]. The latest classification by the World Health Organization (WHO) from 2020 distinguishes nearly 100 different entities of malignant soft tissue and bone sarcomas (plus numerous benign tumors and entities with unspecified, borderline or uncertain behavior) [5].

Based on molecular characteristics, sarcomas have been broadly classified into two main subgroups: sarcomas with a complex, unbalanced karyotype and those with a relatively simple, near-diploid karyotype (for review, see [6]). Especially the latter is often associated with recurrent, chromosomal translocations. Due to the simplicity of the involved karyotype, many of them have been recognized already decades ago by cytogenetic analyses; however, over the past few years, next-generation sequencing (NGS) studies led to the detection of numerous additional translocations. Among all WHO-defined soft tissue and bone sarcomas [5], including the more recently defined soft tissue sarcoma entities pericytoma, angiofibroma of soft tissue and biphenotypic sinonasal sarcoma, gene fusions were detected in more than 40 entities [7]. Most of them are associated with a whole group of structurally different but functionally similar fusion proteins, adding another level of complexity to sarcoma biology (Figure 1). In total, more than 150 recurrent fusion proteins have been identified so far, which define the “fusiome” of sarcoma.

The translocations are of great interest both from a diagnostic as well as from a therapeutic perspective.

In diagnostics, translocations are very useful molecular markers for tumor classification. Since the different fusion proteins expressed are mostly very tumor-type specific (pathognomonic), IHC- (see Table 1 for specific markers), PCR-, FISH- or sequencing-based detection of the gene fusions became a routine clinical practice to support histological classification. Moreover, identification of novel translocations is a driver for further sub-classification of individual entities that was not possible with classical pathological methods alone.

In the long run, the translocations and other molecular characteristics, together with associated therapeutic options, might be used for establishment of a novel classification scheme of sarcoma. Such a classification could complement the current histology-based classification and might be helpful for drug selection in the context of precision medicine. Hence, in an approach to classify sarcoma according to the oncogenic mechanism of the involved fusion protein (kinases, epigenetic complexes, transcription factors), we summarize the available literature describing mechanistic aspects of sarcoma fusion proteins in this narrative review.

## 2. Fusions of Kinases

Kinases are the most frequently mutated genes in cancer. In general, the mutations lead to activation of the involved kinase and the corresponding downstream signaling pathways. In the case of fusion proteins, the kinase domain of the translocated kinase is normally fused to a dimerization or oligomerization domain from the fusion partner, leading to self-association and activation of the kinase domain and subsequent downstream signaling. In addition, the fusion partner often contributes with a strong promoter to the oncogenic potential of the highly expressed translocation product. Interestingly, nearly all sarcoma types that contain a kinase fusion protein belong to the fibroblastic/myofibroblastic spectrum of sarcoma (Figure 1), suggesting that the fibroblastic lineage is specifically amenable to transformation by aberrant activation of signaling pathways.

Importantly, the involved kinase is a potential target for therapy. Indeed, treatment with kinase inhibitors is initially often effective. However, clinical experience gained during the last decade with a range of kinase inhibitors has shown that application as single agents will lead to the development of drug resistance in most cases. Prevention or circumvention of resistance mechanisms, therefore, remains a major goal for the future. Combination therapies with standard-of-care chemotherapy, other targeted drugs or immunotherapy are interesting options in this context [22].

### 2.1. ALK/ROS1/PDGFRβ Translocations in Inflammatory Myofibroblastic Tumors and Leiomyosarcoma

Kinase fusions are found in the majority of inflammatory myofibroblastic (IMF) tumors [23]. In 50–70% of all cases, translocations involving the receptor tyrosine kinase ALK are found, while in ALK-negative cases, translocations of other receptor tyrosine kinases, including ROS1, PDGFRβ and NTRK3, have been identified [23,24,25,26]. Numerous ALK fusion partners have been identified in IMF tumors so far, the most frequent one being tropomyosin TPM3. With the exception of Fibronectin1 (FN1)::ALK fusions (see also similar FN1::FGFR1 translocations described below), chimeric proteins lack the extracellular and transmembrane ALK domains and contain only the C-terminal kinase domain, while the translocation partner contributes with a strong promoter and a dimerization or oligomerization domain, leading to high expression and ligand-independent oncogenic activation of the ALK kinase [26]. Besides IMF, ALK fusions have also been detected in a small subset (<2–5%) of leiomyosarcoma involving a range of different fusion partners such as KANK2 and ACTG2, two proteins highly expressed in smooth muscle, and STK32B, IGFBP5, and TNS1 [27]. Furthermore, ALK activation by different types of mutation are also found outside of the sarcoma family, including neuroblastoma or anaplastic thyroid cancer and others, and it has been suggested to group all of these tumors under the term “ALKomas” [28], an early attempt to classify tumors according to their molecular characteristics with therapeutic implications.

Indeed, all of these fusion proteins are potential targets for therapy with kinase inhibitors. Several ALK inhibitors are FDA-approved, and promising clinical effects with kinase inhibitors against ALK and ROS1 have been detected in several IMF tumor cases [23,29,30,31].

### 2.2. NTRK Translocations in Congenital and Adult Fibrosarcoma

A majority of infantile fibrosarcomas (IF, sny: congenital fibrosarcoma) are associated with ETV6::NTRK3 (TrkC) translocations [32,33], while in rarer cases, related translocations involving NTRK1,-2, and, -3 with different fusion partners have been identified [34,35,36]. Similar STRN(3)::NTRK3 translocations have recently also been detected in some adult fibrosarcomas [37]. NTRK1-3 are receptor tyrosine kinases of the tropomyosin-related kinase (TRK) family involved in neuronal development and are, therefore, abundantly expressed in the nervous system; however, their expression spectrum clearly goes beyond the nervous system [38]. The prototypic fusion partner ETV6 belongs to the ETS family of transcription factors. The ETV6::NTRK3 chimeric protein contains the sterile alpha motif (SAM) oligomerization domain of ETV6 and the tyrosine kinase domain of NTRK3 and lacks the DNA binding domain of ETV6 [39]. Formation of higher order polymers through self-association of the SAM domain leads to activation of both the NTRK-kinase and connected downstream signaling pathways including RAS-MAPK and PI3K-AKT via the adapter protein IRS1 [39,40,41]. The fusion protein has transformation abilities in different cell lineages [39]. Importantly, a variety of similar as well as alternative NTRK fusions are found in a range of tumors outside of the sarcoma field, including different brain tumors but also carcinoma and even some leukemias, albeit in most of the affected entities with a low frequency (for review, see [42]). In analogy to the “ALKomas”, these tumors could be subsumed as “NTRKomas” and NTRK-directed therapies might be of benefit in all of these cases. Indeed, the treatment of NTRK fusion-positive cancers with TRK inhibitors showed impressive response rates, and larotrectinib was recently FDA-approved as the first “tumor-agnostic” drug for the treatment of NTRK fusion-positive tumors [43,44]. In the case of pediatric patients, however, long term side effects of treatment with NTRK inhibitors, especially in the CNS, have to be carefully ascertained in the future.

### 2.3. BRAF Fusions in Myxoinflammatory Fibroblastic Sarcoma, Myxofibrosarcoma and Infantile Fibrosarcoma

BRAF fusions have been detected in different sarcomas of the fibroblastic/myofibroblastic arm of sarcoma. Single studies detected BRAF fusions in 22 percent of myxoinflammatory fibroblastic sarcoma (MIFS) (mostly TOM1L2::BRAF and one single ROBO1::BRAF), a SLC37A3::BRAF fusion in a single case of myxofibrosarcoma and SEPT7::BRAF and CUX1::BRAF fusions in tumors with some characteristics of infantile fibrosarcoma [36,45,46,47]. BRAF fusions lack the N-terminal autoinhibitory domain and only retain the C-terminal kinase domain [45]. Interestingly, the equally frequent translocation t(1;10) found in MIFS, giving rise to *TGFBR3::MGEA5* fusions, does not generate a functional fusion protein but instead has been linked to upregulation of *FGF8* expression, which is located near the *MGEA5* locus. Both BRAF fusions and FGF8 overexpression lead to increased activation of the ERK1/2 pathway, suggesting that this signaling axis is of special relevance in MIFS [45,48].

Since BRAF is activated by point mutations (most frequently BRAF V600E) and translocations in a large number of tumors also outside the sarcoma field [49], it has been in focus as a target for therapy for quite a while. A prominent example in this context is melanoma. However, while RAF inhibitors have improved survival of patients with melanoma, their effectiveness is limited by the development of resistance by various mechanisms [49]. Hence, a better understanding of these mechanisms in combination with innovative RAF inhibitors is necessary to improve the performance of this approach.

### 2.4. FGFR1 Translocations in Phosphaturic Mesenchymal Tumor

Phosphaturic mesenchymal tumors (PMTs) are mostly benign tumors; however, rare cases of malignant forms have been described [50]. Nearly 50% of PMTs have a FN1::FGFR1 translocation [51,52]. The FN1::FGFR1 fusion proteins contain a large N-terminal part of FN1 and the C-terminal part of FGFR1. Interestingly, the breakpoint in FGFR1 is in the extracellular domain, and the fusion proteins are expected to retain the FGF binding domains [51,52]. This is in sharp contrast to FGFR fusions found in other tumors where the breakpoint normally lies in the intracellular domain of FGFR, leading to fusion of only the kinase domain to the partner protein. However, structurally similar fusions of FN1 with ALK have been found in ovarian cancer and inflammatory myofibroblastic tumors (IMFT) (see above) [23,53]. This suggests that FN1 kinase fusion proteins are only active when incorporated into the plasma membrane. The fibronectin part of the fusion includes domains involved in fibronectin self-assembly and fibrin formation and, therefore, might be involved in ligand-independent activation of the FGFR1. However, in PMT also, ligand-based activation of FN1::FGFR1 might play a role. This would explain the high amount of FGF23 that is produced by this type of tumor and which leads to the characteristic osteomalacia (bone softening) via renal phosphate wasting associated with this malignancy [54]. Hence, FGF23 might be involved in an autocrine mechanism necessary for full activation of the fusion protein [52]. For efficient binding of FGF23 to FGFRs in vivo, Klotho seems to be necessary as co-receptor, a mechanism by which the tissue specificity (particularly in the kidney) of this circulating peptide hormone is achieved under normal circumstances. Whether Klotho also plays a role in activation of the FN1::FGFR1 fusion protein by FGF23, however, needs further clarification.

Interestingly, an alternative FN1::FGF1 fusion is present in a smaller number of PMTs [52]. This fusion contains most of the FGF1 protein and, therefore, potentially acts like normal FGF1 as secreted, aberrant growth factor (similarly to the PDGFB fusions described below).

## 3. Fusions of Growth Factors

This type of fusion protein involves a ligand for a receptor tyrosine kinase. The translocation partner mainly contributes with a strong promoter, leading to high expression levels of the ligand, which acts in an autocrine manner and stimulates the signaling pathway downstream of the receptor. Interference with the activity of the involved receptor, therefore, represents a suitable potential therapeutic approach in this case.

### PDGFB/D Translocations in Dermatofibrosarcoma Protuberans

More than 90% of all cases of dermatofibrosarcoma protuberans (DFSP) contain a translocation of the Collagen alpha-1(I) chain (COL1A1) with PDGFB, often in a supernumerary ring chromosome. In rarer cases, all involving tumors arising in the breast of females, a COL6A3::PDGFD fusion has been detected [55]. The COL1A1::PDGFB fusion transcript is translated to form a precursor fusion protein that is further processed into a mature and fully functional PDGFB protein, which serves as ligand for PDGFRB [56]. The aberrant fusion proteins, therefore, probably cause autocrine PDGFR pathway stimulation and cell proliferation.

Different clinical studies of DFSP with the PDGFR inhibitor Imatinib showed dramatic effects in advanced cases and led to the introduction of systemic Imatinib treatment into clinical practice for DFSP [57,58,59,60,61].

## 4. Fusion Proteins Affecting Transcription

Transcriptional dysregulation is one of the most fundamental hallmarks of cancer [62,63]. While also fusion proteins acting at the level of signaling pathways, as described above, ultimately will mediate some of their effects via changes of gene expression (or alternatively need co-dysregulation of transcription by other mechanisms for full malignant transformation [63]), the fusion proteins described below do so by directly affecting gene expression at the level of the chromatin. Fusions of TFs and epigenetic regulators both belong to this category. In general, TFs and epigenetic regulators tightly collaborate to orchestrate gene expression. TFs bind to specific DNA motifs via DNA binding domains and control the specificity and timing of gene expression. In contrast, epigenetic regulators are usually part of larger complexes, which, after recruitment to the chromatin by TFs, regulate DNA accessibility. We thus categorized fusion proteins with a DNA binding domain as fusion TFs, while fusion proteins that do not directly bind to DNA but are part of an epigenetic complex are defined as epigenetic fusion proteins (Figure 1).

An interesting open question is whether such transcription-driven cancers are generally more sensitive towards interference with transcriptional regulation than signaling-driven cancers [62].

### 4.1. Fusions of Components of Epigenetic Complexes

An important level affected in cancer that leads to transcriptional dysregulation is the level of epigenetics. Epigenetic regulators are among the most frequently mutated genes in cancer, affecting numerous cancer subtypes [64,65]. Driver mutations affect all types of epigenetic regulators. Importantly, the reversibility of the epigenetic modifications opens promising options for therapeutic interventions. Epigenetic enzymes are prime targets for such an approach; however, also some reader domains like bromo- and chromodomains contain druggable cavities for small molecules. Different inhibitors directed against members of the epigenetic machinery are in clinical development for the treatment of a range of cancer types [66].

**Figure 2:**
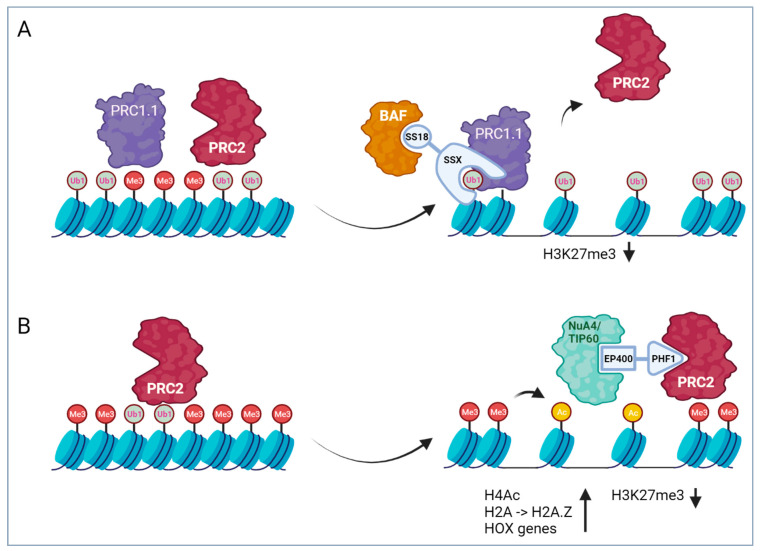
Scheme depicting the mechanism of action of fusion proteins acting as components of large epigenetic complexes in sarcoma. (**A**) Mechanism of action of SS18::SSX in synovial sarcoma. The SSX part of the fusion proteins binds to H2AK119Ub1 sites in Polycomb domain regions of the genome, directing the BAF complex to these regions and antagonizing Polycomb activity. (**B**) Mechanism of action of EP400-PHF1 in OFMT. The fusion induces formation of a megacomplex composed of NuA4/TIP60 and PRC2, antagonizing PRC2 activity in Polycomb domains. The net effect of both fusion proteins is a disturbance of the balance between the repressive action of Polycomb complex 1/2 (PRC1/2) (chromatin compaction) and the activating activity of chromatin remodelers (chromatin decompaction). This affects DNA accessibility and leads to activation of gene expression. Image created with BioRender.

Fusion proteins involved in epigenetic regulation are found in several sarcoma entities, including synovial sarcoma (SS), ossifying fibromyxoid tumors (OFMT), endometrial stromal sarcomas (ESS) and different entities of the group of undifferentiated small round cell sarcomas of soft tissue and bone (previously called Ewing-like sarcomas (ELS)). Interestingly, most of these entities belong to the group of sarcomas with uncertain differentiation, suggesting that epigenetic dysregulation leads to major dysregulation of gene expression patterns and cell identity. In the case of SS and OFMT, the mechanism of action of involved fusion proteins is quite well understood, while in the other cases it is less clear. Strikingly, it seems that in most cases, an important effect of the involved fusion proteins is a shift in the balance between Polycomb repressor complexes (PRCs) and some ATP-dependent chromatin-remodeling complexes (Figure 2). The two PRCs PRC1 and PRC2 complementarily repress gene expression by ubiquitination of H2AK119 and tri-methylation of H3K27, respectively, which act as repressive chromatin modifications and lead to transcriptional silencing. ATP-dependent chromatin remodeling complexes (CRCs) act as molecular motors that use the energy from ATP hydrolysis to regulate chromatin organization by the assembly, positioning or disruption of nucleosomes. Moreover, these large complexes also contain numerous other proteins with various epigenetic functions, including histone modification activities (reader, writer and eraser), which act in concert to affect chromatin accessibility. The antagonistic relationship between repressive PRCs and the chromatin remodeler SWI/SNF was recognized in *Drosophila melanogaster* already two decades ago by studying their effect on the expression of *Hox* genes during embryogenesis [67,68]. Together with a broad range of other proteins that counterbalance the effect of PRCs during *Drosophila* development, SWI/SNF belongs to the trithorax (TrxG) proteins. The fusion proteins found in sarcoma seem to interfere with the tight physiological balance between PRCs and CRCs, leading to induction of a tumorigenic gene expression program.

#### 4.1.1. SS18 Translocations in Synovial Sarcoma

Nearly 100 percent of synovial sarcomas (SSs) have translocations involving the SS18 component of the BAF (mammalian SWI/SNF) chromatin remodeler complexes or, in rare cases, the homologue protein SS18L1 (CREST) [69,70,71,72]. Different BAF components are mutated in more than 20% of all cancers [73], highlighting that functional aberration of this complex is pro-tumorigenic in many types of cells. Interestingly, the different mutations are highly tumor type-specific. In SS, the fusion partners of SS18 are proteins from the SSX family, SSX1, SSX2 and, in rare cases, SSX4. The highly homologous SSX proteins (10 members) are classified as cancer testis-antigens since their expression is restricted to adult testes and different types of cancer, and have been associated with transcriptional repressor functions [74]. In all known SS18::SSX fusion proteins, the last eight amino acids of SS18 are replaced by the C-terminal 78 amino acids of the SSX proteins containing the SSXRD domain that has been shown to be involved in interaction with PRCs [75,76].

It has been suggested that the general mechanism of oncogenesis involved in BAF mutant cancers is related to the ability of BAF complexes to oppose PRC-mediated repression [77]. Along these lines, an important aspect of the function of SS18::SSX is its binding to the BAF complexes, outcompeting wildtype SS18 and, in addition, displacing the BAF component SMARCB1 (INI1, BAF47) from the complexes [78]. The aberrant complex is then retargeted from enhancers to broad Polycomb domains to oppose PRCs and activate bivalent genes [79].

Recruitment of the aberrant complex to the chromatin depends on the interaction of the SSX tail with H2AK119Ub1 sites, which are deposited by PRC1 (in the SS cells, the variant Polycomb repressive complex 1.1 (PRC1.1) seems to be most relevant [80]) in an early step of Polycomb domain formation [79,80]. At the same time, SSX stabilizes PRC1.1 complex presence and thereby further potentiates H2AK119Ub1 deposition and binding of additional oncogenic complex [80] (Figure 2). In rare SS cases, alternative partners have recently been found to be fused to SSX1, including EWSR1 and MN1 [81]. These fusions might be able to recruit also other transcriptional regulators to Polycomb domains, like p300 in the case of MN1::SSX1, which thereby induces the deposition of H3K27Ac [80]. This suggests that the SSX tail is the main determinant conferring specificity to the fusion protein.

In general, this gain-of-function effect of the SS fusion proteins contrasts with the effect of BAF loss-of-function mutations found in other tumors, such as the bi-allelic loss of *SMARCB1* (95%) or *SMARCB4* (5%) found in all malignant rhabdoid tumors (MRT) or in a majority of epithelioid sarcomas (EpS) [82,83,84]. There, the loss of BAF activity leads to aberrantly high PRC2 activity at tumor suppressor genes such as *CDKN2A* and others and their transcriptional silencing [85,86]. In such cases, both preclinical and clinical data have demonstrated that PRC inhibition is effective against tumor growth [87,88,89]. Based on these findings, the EZH2 inhibitor tazemetostat has recently been FDA-approved for the treatment of advanced *SMARCB1*-deficient EpS. In contrast, the oncogenic mechanism of action of the fusion proteins in SS suggests that inhibition of the oncogenic BAF complex might be a more effective therapeutic approach here. Since SS18::SSX is a challenging drug target, other BAF components like SMARCA2/4 (bromo- or ATPase domain) or ARID1A/B (E3 ligase activity) or PRC1.1 components might be targeted [76,90].

#### 4.1.2. PHF1 Translocations in OFMT and Endometrial Stromal Sarcoma (ESS)

Up to 85% of the cases of OFMT have translocations involving PHF1 [91]. PHF1 belongs to the Polycomb-like (PCL) protein family and acts as an accessory component of PRC2. It contains different domains for interaction with chromatin, including a Tudor domain that interacts with H3K36me3/2 found in gene bodies of actively transcribed genes, but also two PHD domains and a winged helix (WH) domain, which binds to DNA [92,93,94]. PHF1 contributes to PRC2 activity in different ways. While binding to H3K36me3/2 has been found to inhibit HMTase activity of associated PRC2, binding to DNA via the WH domain has the opposite effect on PRC2 [94,95]. Interestingly, PHF19, a homologue of PHF1, has been shown to recruit some histone demethylases capable of demethylating H3K36me3 [96,97]. Taken together, these findings hint towards a model in which PHF1 (and PHF19) mediates targeting and/or spreading of PRC2 to H3K36me3-marked loci in the genome, followed by H3K36me3 demethylation and subsequent PRC2-mediated tri-methylation of H3K27 [98]. Overall, these chromatin modifications then lead to de novo transcriptional silencing of the involved genes.

The fusions in OFMT usually contain the whole coding region of PHF1. In roughly half of all OFMT cases, the fusion partner of PHF1 is EP400 (EP400::PHF1) [91,99,100], while in rarer cases, MEAF6::PHF1, EPC1::PHF1, and PHF1::TFE3 fusions have been detected [91,101]. Interestingly, EP400, MEAF6 and ECP1 are all components of the NuA4/TIP60 complex, a hybrid complex composed of homologues of the yeast NuA4 and SWR1 complexes. NuA4/TIP60 belongs to the MYST-family acetyltransferase complexes and is composed of at least 16 subunits with at least three enzymatic activities [102]. In addition to the H4/H2A acetyltransferase, it contains a DNA helicase and an ATP-dependent chromatin remodeling activity mediating H2A-H2A.Z and H3.1-H3.3 histone exchange [103,104]. As an ATP-dependent chromatin remodeler protein of the SWR1 class, EP400 is relevant for this latter function. The NuA4/TIP60 complex is involved in a wide variety of cellular functions, including transcriptional activation, DNA repair, chromosome stability and others.

Hence, the overall picture is that in most cases, the OFMT fusion proteins are composed of an N-terminal NuA4/TIP60 component and a C-terminal PRC2 subunit, albeit in rare cases, fusions of other epigenetic regulators have been found, including ZC3H7B::BCOR, CREBBP::BCORL1, KDM2A::WWTR1 and CSMD1::MEAF6 [91,101,105,106]. Interestingly, in low-grade ESS, a range of similar NuA4/TIP60::PRC2 fusions have been detected. In addition to fusions of PHF1 with JAZF1, EPC1/2, BRD8, and MEAF6, the most frequent fusion in ESS is composed of the NuA4/TIP60 component JAZF1 and the PRC2 component SUZ12 (JAZF1::SUZ12) [107,108,109,110,111,112]. Different lines of evidence from both OFMT and ESS demonstrate that the fusion proteins act as a molecular bridge between the two involved complexes and generate a NuA4/TIP60-PRC2 megacomplex [112,113]. This leads to increased histone acetylation and H2A.Z incorporation at PRC2 regulated genes and aberrant induction of expression of silenced genes [113]. This is in line with the finding that ectopic expression of EP400::PHF1, MEAF6::PHF1 or PHF1::TFE3 in human fibroblasts led to upregulation of hundreds of genes, with the most strongly enriched gene set being composed of genes with CpG island promoters that are silenced by H3K27 trimethylation in neuronal progenitors [106]. An important aspect is that this effect is not seen genome-wide at all PRC2 bound regions, but only at specific sites. Hence, in OFMT, global H3K27me3 levels were claimed to be unaffected [106], while in certain topologically associating domains (TADs) such as the TAD containing part of the *HOXD* cluster, the fusion mediates increased histone acetylation and H2A.Z incorporation [113].

#### 4.1.3. BCOR Translocations in Undifferentiated Small Round Cell Sarcomas of Bone and Soft Tissue and OFMT

Translocations involving BCOR have been detected in different types of sarcoma such as OFMT and entities of the group of undifferentiated small round cell sarcomas (URCS), which in the past, also because of their CD99 positivity and other similarities with Ewing sarcoma (EwS), have been termed as “Ewing-like sarcoma”. Interestingly, some of the fusions have been found in both OFMT and URCS, and fusion proteins with BCOR as N- and C-terminal partner exist. In URCS with *BCOR* alterations, the most frequent fusion is BCOR::CCNB3; however, also BCOR::MAML3, KMT2D::BCOR and ZC3H7B::BCOR have been described [114,115]; for review, see [116]. The latter fusion protein as well as the similar protein ZC3H7B::BCORL1 have been found in OFMT [91,101]. Strikingly, a subgroup of URCS tumors with BCOR alterations contain a mutant BCOR form with an internal tandem duplication of a region towards the C-terminal end [117], a mutation that is also found in a range of other tumor types like clear cell sarcoma of the kidney and others [118]. The fact that all of the different BCOR-rearranged sarcomas cluster together in gene expression profilings suggests that all involved mutant BCOR proteins have a common mechanism of action [119].

BCOR (or its close homologue BCORL1) is a component of the non-canonical PRC complex PRC1.1. Like the canonical PRC1 complex, non-canonical PRC complexes contain a RING1A or RING1B E3-ubiquitin ligase necessary for gene silencing [120]. However, they do not contain a component that is able to bind to H3K27me3, which links canonical PRC1 with PRC2 activity, and, therefore, are recruited by alternative mechanisms to the chromatin. PRC1.1 binds to non-methylated CpG islands via its component KDM2B [121]. Hence, it seems that like SS, also in BCOR-rearranged tumors, hijacking of PRC1.1 and dysregulation of PRC activity contributes to tumorigenesis. Along these lines, different *HOX* family members were found among the most upregulated genes in a BCOR::MAML3 sarcoma when compared to Ewing sarcoma with EWSR1::FLI1 fusions and CIC::DUX4 positive sarcomas [115]. This raises the important question of whether similar therapeutic targets could be addressed in SS and the different BCOR-rearranged tumors.

### 4.2. Fusions of Transcription Factors

This type of fusion protein is the most frequent one found in sarcoma. In its prototypic version, it contains the DNA-binding domain of a transcription factor (TF) fused to the transactivation domain of a second TF or of a transcriptional co-regulator. Based on this structure, the fusion protein has the ability to de-regulate the target gene spectrum of the first TF (“determinator”) via the aberrant activity of the transactivation domain of the fusion partner (“regulator”). Determinator TFs are often involved in regulation of lineage-specific genes and affect developmental progression and cell fate under normal circumstances. While wildtype determinators are switched off during development by feedback signals activated in later cell stages of the lineage [122], fusion TFs escape from these regulatory mechanisms, leading to a permanently high and uncontrolled expression of the target genes. This freezes cells in a proliferative state and blocks the differentiation process.

Some TFs have the capability to reprogram lineages, which allows conversion of one cell type into a different one [123]. Hence, it is not excluded that some of the fusion proteins reprogram the cell of origin they occur in and induce the expression of a gene signature characteristic for another lineage. This complicates identification of the cell of origin, which is still unknown in most of the cases.

From a therapeutic perspective, TFs are challenging drug targets, as their prototypic structure complicates the development of traditional small molecule inhibitors. The DNA binding domains are structured parts; however, protein–DNA interaction surfaces are normally too large to be efficiently affected by small molecules. In contrast, the transactivation domains are often largely intrinsically disordered and involved in phase separation, a function that is even harder to interfere with. Based on these characteristics, TFs have been claimed to be “undruggable” for a long time. Hence, in the past, indirect ways to interfere with the activity of fusion TFs were a focus of attention. However, a better understanding of their biology, in particular, their interplay with the most relevant cofactors, might reveal novel opportunities for inhibition [124].

#### 4.2.1. EWSR1 Translocations in 11 Different Sarcoma Entities

Translocations involving EWSR1 are found in numerous sarcomas, including Ewing sarcoma/PNET, low-grade fibromyxoid sarcoma, sclerosing epithelioid fibrosarcoma, mixed tumor/myoepithelioma, angiomatoid fibrous histiocytoma, clear cell sarcoma, desmoplastic small round cell tumor, extraskeletal myxoid chondrosarcoma, myxoid/round-cell liposarcoma, malignant peripheral nerve sheath tumor and intraosseous rhabdomyosarcoma. EWSR1 contains an N-terminal transactivation domain and a C-terminal RNA-binding domain that is involved in RNA metabolism [125]. Breakpoints slightly differ among the different tumors (exons 7, 8, 12), but in general, the N-terminal transactivation domain is included in the fusion protein, while the C-terminal RNA-binding part is not. Hence, the EWSR1 part acts as regulator in the fusion protein. In some cases, EWSR1 is substituted by FUS (TLS) or TAF15, which have similar characteristics and together build the FET family of proteins. In the different sarcomas, these FET-family proteins are fused to different transcription factors that deliver the sequence-specific DNA-binding domains and act as determinator parts in the fusion proteins. As the N-terminal part of the fusion, the promoter of the involved FET protein drives expression of the fusion protein. Since the FET-family proteins are ubiquitously expressed [126], their highly active promoters allow high expression of the fusion proteins in a broad range of cells. The best studied among all of these fusion proteins is EWSR1::FLI1, found in Ewing sarcoma. EWSR1::FLI1 can exert both activating and repressing functions, and both of these functions are of importance in Ewing sarcoma [127,128]. Recent studies have shed some light on the molecular mechanism behind these two opposing functions. It has been suggested that the repressive function is based on a competition with wildtype ETS transcription factors for binding to single GGAA sites present in regulatory elements in the genome [129]. Displacement of the wildtype TFs by the fusion protein leads to reduced expression of involved target genes. Recruitment of repressive epigenetic complexes like NuRD might be involved [128]. The activating function, in contrast, is based on the ability of the fusion protein to act as pioneer factor at GGAA microsatellite regions (msats) and induce de novo enhancers at these sites [129]. These msats are poorly conserved regions and typically represent condensed chromatin regions in normal tissues. It is unclear why some msats remain inaccessible to EWSR1::FLI1, despite its pioneer function. Strikingly, some of the induced genes are neo-genes, which are exquisitely expressed in Ewing sarcoma but never in other cancer entities [130]. The pioneer function of EWSR1::FLI1 is linked to the prion-like domain (PrLD) of the EWSR1 part, which is able to form phase-separated compartments and recruit different chromatin-modifying complexes, including BAF, p300 and mixed-lineage leukemia complexes, to these sites that ultimately remodel the chromatin and induce gene expression [129,131]. PrLDs were identified in 242 human proteins [132]. Initially reported in the context of neurodegenerative disorders, their role in oncogenic mechanisms is a growing field. These low-complexity domains are enriched in glycine and uncharged polar amino acids, including glutamine, asparagine, tyrosine, and serine. PrLDs containing proteins contribute to the formation of dynamic, membraneless organelles through liquid–liquid phase separation (LLPS) and are involved in various cellular processes, such as transcriptional regulation and RNA metabolism. The FET-family proteins (including FUS, EWSR1, and TAF15) contain a PrLD that is conserved in the FET-translocated sarcomas, including in Ewing sarcoma. In EWSR1::FLI1, PrLD-mediated phase-separation events depend on tyrosine residues necessary for phase transitions [131,133]. Subsequently, the PrLD interacts with the BAF complex, promoting target gene activation. The ability of PrLDs to facilitate the recruitment of chromatin remodeling complexes, as demonstrated in Ewing sarcoma, could be applicable to other sarcoma types. For instance, similar mechanisms may occur in other FET-fused sarcomas or MAML3- or NCOA2-fused sarcoma, which also contain PrLD domains [134]. Similarly, the SS18 low-complexity QPGY domain, conserved in the SS18::SSX chimeric protein in synovial sarcoma, enables the recruitment of BRG1 into LLPS [135]. Moreover, most eukaryotic transcription factors (TFs) also contain intrinsically disordered low-complexity sequence domains (LCDs) that contribute to LLPS-associated mechanisms present at transcriptional hubs [136,137]. These LCDs are also partially conserved in chimeric oncogenes and may, therefore, exert a significant impact on transcriptional regulations and LLPS mechanisms. By targeting the PrLD- or LCDs-mediated LLPS mechanisms, it might be possible to interfere with the oncogenic transcriptional activity in these sarcomas. These strategies could be explored as a therapeutic avenue by hindering the formation of tumor-specific enhancers and impeding the expression of oncogenic target genes. Additionally, exploring the potential of displacing LLPS into the nucleolus, as demonstrated with EWSR1::FLI1, could be a strategy to sequester oncogenic fusion proteins, inhibiting their transcriptional functions [133,138].

Overall, it is reasonable to speculate that these functional properties of EWSR1 may serve as the basis for its widespread involvement in sarcoma tumorigenesis. Furthermore, the neomorphic functions of the fusion proteins probably contribute to the extensive transcriptional dysregulation detected in these sarcomas, impeding clear lineage association and, as a result, classification of many of these sarcomas to the group of sarcomas with uncertain differentiation (Figure 1).

#### 4.2.2. CIC Translocations in Undifferentiated Small Round Cell Sarcomas of Soft Tissue and Bone

**Figure 3:**
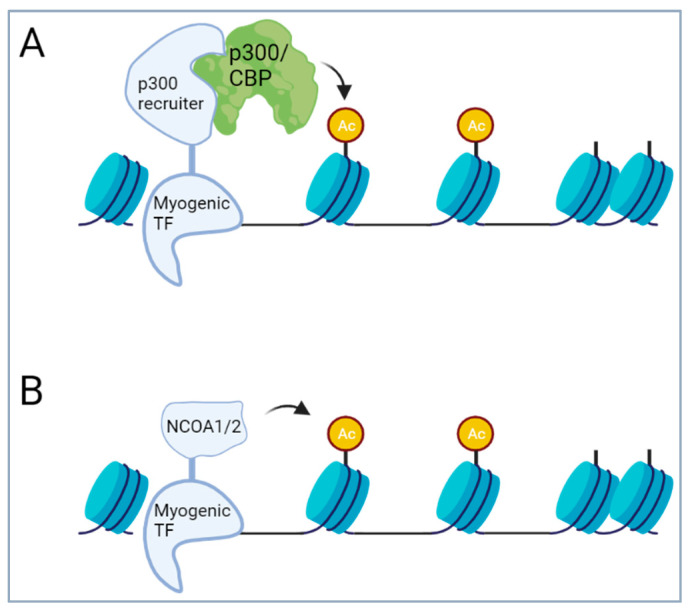
Recruitment of histone acetyltransferase (HAT) activity by fusions involving myogenic transcription factors is a common theme in different muscle-related sarcomas. (**A**) Fusion of myogenic TF with p300/CBP recruiters like FOXO1/4 or CITED2. CIC-fusion TFs found in CIC-rearranged sarcomas have a similar mechanism of action, with DUX4, FOXO4 and NUTM1 acting as p300 recruiters. (**B**) Direct fusion of myogenic TF with protein containing HAT activity like NCOA1/2. Image created with BioRender.

Translocations involving the transcriptional repressor CIC have been recently described in some undifferentiated small round cell sarcomas of soft tissue and bone. In these sarcomas, the transcriptional repressor CIC is fused to the double homeobox transcription factor DUX4 or to the homologue DUX4L10, or in rare cases, to FOXO4 or NUTM1 [139,140,141,142]. DUX4 is a retrotransposed gene that is located within a D4Z4 repeat array in the sub-telomeric regions of chromosomes 4 and 10. These arrays typically contain from 11 to more than 100 repeats. Each D4Z4 repeat contains one copy of the DUX4 gene; however, only the most telomeric gene is expressed, while the other genes are silenced by hypermethylation. DUX4 from both chromosomes 4 and 10 has been found to be involved in the translocations with CIC. The fusion proteins are composed of a large part of CIC, including its HMG box DNA binding domain and a small C-terminal part of DUX4 (or other fusion partners), and have been shown to transform NIH3T3 fibroblasts [139]. The small DUX4 part binds and recruits p300/CBP, converting the CIC repressor into a transcriptional activator. The DUX4 part has been shown to increase transcriptional activity of CIC by more than 100-fold in reporter assays [139,143]. In cells, the fusion induces H3K27 acetylation and expression of a distinct gene expression signature [143,144]. Importantly, the primary function of the alternative fusion partners FOXO4 and NUTM1 is also p300/CBP recruitment [145]. Overall, these data suggest p300/CBP recruitment followed by hyperacetylation is the main effect of the fusion protein (Figure 3) and that blocking p300/CBP might represent a way to interfere with CIC::DUX4 activity for therapeutic purposes [143].

Interestingly, in a recently described sarcoma case, a NUTM2A::CIC translocation was described [146]. In this case, the DNA binding domain of CIC was not present in the fusion protein. Instead the C-terminal part with the two capicua homology domains was fused to the N-terminus of NUTM2A.

#### 4.2.3. Translocations of Transcription Factors of the Skeletal Muscle Lineages

Myogenic differentiation is a complex process that is under the control of a cascade of transcription factors. The process is initiated by the upstream regulators sine oculis-related homeobox 1 and 4 (Six1 and −4), followed by activation of PAX3 and PAX7, which direct expression of the downstream myogenic regulatory factor (MRF) family (Myf5, MyoD, Myogenin and Myf6 (Mrf4)).

Numerous TFs involved in myogenic differentiation are part of translocations found in different tumors with myogenic features. Fusions of PAX3 and −7 are the drivers of both alveolar rhabdomyosarcoma (aRMS) and biphenotypic sinonasal sarcoma (SNS) [147]. In more than 80% of aRMSs, the DNA binding domains of PAX3 or −7 (a paired box and a homeobox domain) are fused to the transactivation domain of FOXO1. Rare variant fusion partners of PAX3 include FOXO4, NCOA1, NCOA2, INO80D and GLI2 [148,149,150,151,152]. With the exception of INO80D, these partner proteins are all transcription factors or transcriptional co-regulators. INO80D is a component of the INO80 chromatin remodeling complex. The INO80 complex belongs to the same subgroup of chromatin remodeling complexes as the Nu4A/TIP60 complex described above and has diverse functions, including activation of transcription [153].

Rearrangement of the *PAX3* locus has also been found in a majority of SNS cases (89–96%). In more than half of the cases, a fusion of PAX3 with MAML3, a transcriptional coactivator of the Notch-intracellular domain, is involved [154,155], while in some of the remainder, PAX3 is fused to FOXO1 or NCOA1, revealing some similarity with aRMS [154].

Another set of fusions found in myogenic tumors involves serum response factor (SRF). SRF is a widely expressed TF that has also been associated with cardiac, smooth muscle (SM) and skeletal muscle function [156,157]. Interestingly, SRF serves as platform for two antagonistic families of co-factors [158]. One family is composed of the three ternary complex factors (TCFs) ELK1, NET, and SAP1, while the other one comprises the MYOCARDIN-related transcription factors (MRTF). In SM cells, SRF together with TCFs stimulates a pro-proliferative expression program, while together with MRTFs, in particular MYOCARDIN, it induces expression of genes associated with myogenesis. Based on this molecular switch, SM cells show a high degree of phenotypic plasticity and can change between more differentiated and proliferative stages, highlighting SRF as a central regulator of cell fate [159]. Fusions of SRF have been detected in different tumors of the muscle lineage. Fusions between SRF and NCOA2 were detected in some spindle cell rhabdomyosarcomas, while fusions between SRF and NCOA1 or FOXO1 were recently found in “well differentiated” RMS [160,161,162]. Furthermore, fusions between SRF and RELA or ICA1L have recently been identified in some tumors with a smooth-muscle-like phenotype [163,164].

Finally, in spindle cell rhabdomyosarcomas, fusions TEAD1::NCOA2 and VGLL2::NCOA2/CITED2 have been described. While TEAD1 is a TF, known for its involvement in the Hippo pathway as partner for YAP/TAZ, VGLL proteins are transcriptional co-activators. Both TEAD1 and VGLL2 have been associated with functions in the muscle lineage [165,166]. Interestingly, VGLL proteins are known co-factors of TEAD1 [167,168], suggesting that the different fusions are involved in the same pathways.

Overall, it is striking that in the different myogenic tumors, the translocated myogenic TFs are fused to a relatively small set of similar fusion partners. The recurrent involvement of FOXO1 and NCOA1/2 strongly indicates a common mechanism of action. Importantly, NCOA1/2 contain a HAT activity [169]. Recruitment of p300/CBP leading to strong H3K27 acetylation has also been shown to play a major role in the activity of both wildtype FOXO proteins as well as PAX3::FOXO1 fusion proteins in aRMS [170,171]. Finally, also the co-activator CITED2 (Cbp/p300-interacting transactivator 2) has been described to link transcription factors like TFAP2 with p300/CBP [172]. Overall, this strongly suggests that recruitment of HAT activity to enhancer regions represents a common mechanism of action of many of the myogenic fusion proteins described above (Figure 3). This is remarkably similar to the fusions in the CIC-rearranged sarcoma, suggesting that HAT recruitment is a common theme for fusion TFs in sarcoma. Hence, in all of these cases, interfering with aberrant HAT activity might be a therapeutic option. Importantly, at least in aRMS, global inhibition of p300/CBP does not induce cell death, suggesting that the interaction between the fusion protein and p300/CBP must be selectively blocked for efficient anti-cancer effects [170].

#### 4.2.4. Translocations Activating the Notch Pathway in Mesenchymal Chondrosarcoma and Malignant Glomus Tumors

The Notch pathway is one of the major developmental pathways and as such, involved in control of cell fate and differentiation. Notch is a transmembrane receptor for ligands of the Delta/Serrate/Lag-2 (DSL) family of proteins. Classical Notch signaling is activated upon binding of Notch to ligands present on neighboring cells. Ligand binding initiates two proteolytic cleavage events in Notch, first by ADAM10 in the extracellular juxtamembrane domain, followed by the γ-secretase complex within the transmembrane domain, leading to release of the Notch intracellular domain (NICD). This domain acts as transcriptional co-regulator by binding to DNA-bound repressor RBPJ (CSL) and recruiting co-activators of the mastermind family (MAML1-3) [173], leading to induction of specific target genes, among them *HEY1* and *HES*.

In more than half of malignant glomus tumors (GTs), fusions of Notch-1, -2 and -3 with the mir143 locus have been detected [174]. Breakpoints lie either in the extracellular juxtamembrane region of Notch, as in case of Notch-1/2, or in the intracellular juxtamembrane domain, as in case of Notch-3 [174]. Due to the very strong promoter of mir143 in the affected cells, this translocation leads to high expression of a truncated form of Notch that is nearly identical to the NICD [174]. At least in the cases where the fusion protein contains the transmembrane domain of Notch, it might still be activated by γ-secretase, and corresponding inhibitors might represent an attractive therapeutic option.

Another type of Notch-pathway activation is found in the majority of mesenchymal chondrosarcomas (MCs). There, the Notch pathway effector HEY1 is fused to NCOA2. The HEY1::NCOA2 fusion protein consists of the N-terminal basic helix-loop-helix (bHLH) DNA-binding/protein dimerization domain from HEY1 and the two C-terminal transcriptional activation domains (AD1/CID and AD2) provided by NCOA2. No molecular analyses have been performed yet to study the mechanisms of HEY1::NCOA2 function. However, the structure of the protein suggests that constitutive activation of a HEY1 gene expression signature is involved in the tumorigenesis of MC. Both Notch and HEY1 have been shown to be involved in chondrogenic differentiation of bone marrow-derived stem cells in vitro [175,176]. For terminal differentiation, however, Notch signaling must be downregulated [176]. Failure of downregulation, therefore, might keep the chondrosarcoma cells in a primitive and proliferative state.

#### 4.2.5. NAB2::STAT6 in Solitary Fibrous Tumor/Hemangiopericytoma

The NAB2::STAT6 fusion protein is found in 100% of solitary fibrous tumors (SFTs) [177,178]. Wildtype NAB2 protein acts as transcriptional co-repressor of the TF EGR1. For this function, it contains an N-terminal EGR1-interaction domain and a C-terminal transcriptional repression domain. Since its expression is induced by EGR family members, it acts in a negative feedback loop, keeping EGR1 activity in check. Breakpoints involved in the fusion with STAT6 are located in the C-terminal part, leading to variable truncations of the repressor domain [177]. STAT6 is a TF of the signal transducer and activator of transcription (STAT) family that transmits signals from cytokine receptors to the nucleus, where it induces gene expression. STAT6 is specifically activated by IL-4 and IL-13. Activation of cytokine receptors first leads to activation of associated Janus kinases (JAKs) and phosphorylation of specific sites in the receptor. These sites then serve as docking sites for proteins of the STAT family via their SH2 domains. Recruited STATs are then phosphorylated by the associated JAKs. Phosphorylated STATs homodimerize and move to the nucleus to activate gene expression at specific sites. NAB2::STAT6 fusion proteins found in SFT contain a variable part of the C-terminus of STAT6 that at least contains the transactivation domain. Hence, the fusion protein can bind to EGR1 via the NAB2 part and induces the expression of EGR1 target genes via the STAT6 part [177]. Thereby, the negative feedback mechanism that is mediated by wildtype NAB2 is changed into a positive feedforward loop, leading to permanent high expression of the EGR1 target gene signature [177]. Interestingly, some of the fusion variants were found to correlate with some clinicopathological features [179,180,181]. Among the downstream effectors are different kinases that have been suggested to be potential targets for therapy [177]. However, further studies are necessary to clarify this in more detail in the future.

## 5. Fusions of Transcription Factors with Non-Transcriptional Regulators

A variant of the classical TF fusion proteins is represented by fusions of a transcription factor with a protein that has not been associated with transcriptional regulation. The fusion partner might have a yet-unrecognized role in transcription or might affect the transcriptional activity of the partner TF by an aberrant mechanism.

### 5.1. TFE3 Translocations in Three Different Sarcoma Types

TFE3 is a transcription factor that has been found in translocations in two different types of sarcomas, including alveolar soft part sarcoma (ASPS) and epithelioid hemangioendothelioma (EHE), as well as in perivascular epithelioid cell neoplasms (PEComa), which are neoplasms with uncertain malignant potential [182,183,184]. TFE3 is a member of the microphthalmia transcription factor-transcription factor E (MiTF-TFE) basic helix–loop-helix leucine zipper transcription factor family. In almost all ASPS, it is fused to ASPSCR1 [182]. ASPSCR1 is involved in intracellular trafficking of the glucose transporter GLUT4 and has not been linked to transcription yet [185]. It binds to GLUT4 through its N-terminal part, while the C-terminal part binds the Golgi proteins PIST and GOLGIN-160, thereby trapping GLUT4 storage vesicles in the cytoplasm by linking them to the cis-Golgi matrix [186]. The ASPSCR1::TFE3 fusion protein contains the N-terminal half of ASPSCR1 containing two Ubiquitin-like domains and the C-terminal part of TFE3. Two different breakpoints in the TFE3 gene were found, type 1 and 2, with type 2 containing an additional exon [182]. In both types, the DNA binding domain and nuclear localization signal of TFE3 are included; however, only type 2 also contains the transactivation domain of TFE3. Nevertheless, as determined in reporter assays, both types of fusions have stronger transactivation activities than wildtype TFE3, suggesting that the ASPSCR1 part complements the fusion protein with strong transcriptional activity [187]. In agreement with this finding, it has been shown that the fusion protein functions induce expression of a large target gene signature in ASPS cells [187]. Among these target genes are different druggable proteins that have been suggested to be potential targets for therapy, including MET [188].

Also in PEComas, the TFE3 fusions include its DNA binding domain and nuclear localization signal. The fusion partners in this entity are SFPQ, NONO and DVL2. While SFPQ and NONO are known to be involved in transcriptional regulation, and the fusions with TFE3 might belong to the classical TF class of fusion proteins, DVL2 is not. Instead, it is involved in WNT signaling by binding to the cytoplasmic C-terminus of frizzled family members and transducing the WNT signal to downstream effectors.

Another type of TFE3 fusion is present in some EHEs. While up to 90 percent of all EHEs contain the fusion WWTR1 (better known as TAZ)::CAMTA1, or in a small number of cases, alternative fusions including WWTR1::MAML2 and WWTR1::ACTL6A [189,190], a distinct subgroup comprising 10 percent of all cases contains a YAP1::TFE3 fusion [183]. Both TAZ and YAP1 are effectors of the Hippo pathway, suggesting that this pathway plays an important role in tumorigenesis of EHE. Both TAZ and YAP1 are transcriptional co-activators that lack intrinsic DNA-binding domains and act mainly through TEAD family TFs [191]. The TAZ::CAMTA1 fusion contains the N-terminal part of TAZ including the TEAD binding domain and a large part of CAMTA1, including its transactivation domain. Similarly, in the YAP1::TFE3 fusion, the N-terminal part of YAP1 including the TEAD binding domain is fused to the fourth or sixth exon of TFE3. Both fusions are uncoupled from negative control of upstream Hippo signaling and permanently localized in the nucleus [192,193]. Interaction with TEAD TFs is necessary for tumorigenicity of both fusion proteins and mediates the induction of a transcriptional signature via recruitment of the Ada2a-containing histone acetyltransferase (ATAC) complex [192,194]. ATAC contains GCN5 or the closely related PCAF histone acetyltransferase, which, therefore, represent potential therapeutic targets for EHE.

### 5.2. Translocations Activating Hedgehog Signaling

Pericytoma with t(7;12) with an ACTB (Actin)-GLI1 fusion has been recently defined as a sarcoma entity [195]. These fusion proteins contain an N-terminal part of actin and the C-terminal half of GLI1 including its DNA-binding zinc-finger domains [195]. Similar fusions including MALAT1::GLI1 and PTCH1::GLI1 were recently detected in tumors that resembled pericytic/glomus tumors or myoepithelial tumors [196]. Based on their structure, it can be assumed that the effect of all of these translocations is high upregulation of GLI1 activity by the involved strong promoters and activation of a GLI1 target gene signature. GLI1 is one of three members of the GLI family of TFs, which are effectors of the hedgehog signaling pathway. Aberrant activation of the hedgehog pathway is well known to be tumorigenic in other tumors, including medulloblastoma, basal cell carcinoma and some embryonal RMSs [197,198].

## 6. Fusion Proteins with an Unclear Mechanism of Action

### Trio Translocations Found in Different Sarcomas

Translocations of the kinase TRIO have been recently detected by NGS approaches in different sarcomas with complex karyotypes, including undifferentiated pleomorphic sarcomas, dedifferentiated liposarcomas, pleomorphic rhabdomyosarcoma and myxofibrosarcoma [199,200]. This broad distribution among different sarcoma types might indicate that TRIO fusions are a secondary event implicated in tumor progression [200]. The most frequently detected fusion partner was TERT. It was speculated that the mode of action of the fusion protein is upregulation of telomerase activity [199]. However, the lack of the TERT N-terminal (TEN) domain in the fusion protein, which is essential for telomerase activity [201], argues against this hypothesis [200]. Furthermore, in a small number of cases, alternative out-of-frame fusions of TRIO with LINC0150 and ZNF558 were identified [200]. Taken together, these findings suggest that the truncated TRIO protein instead behaves as an oncogene. TRIO is a multi-domain protein including two guanine nucleotide exchange factor (GEF) domains (with the GEF1 domain activating Rac1 and RhoG, and GEF2 activating RhoA), a SEC14 domain, several spectrin repeats, two SH3 domains, an Ig-like domain, and a serine kinase domain. The breakpoints in the TRIO::TERT fusion protein are all located N-terminal to the GEF2 domain, excluding the kinase domain from the fusion protein. Interestingly, among the different splice variants of wildtype TRIO, two neuronal-specific forms have the exact same exon composition as the one found in the fusion protein. The GEF activity of these isoform(s) has been suggested to be associated with poor patient survival and different physiological parameters, including invasion, migration and proliferation in glioblastoma [202], suggesting that the oncogenic activity of the fusion proteins might also be based on the GEF1 activity.

## 7. Therapeutic Perspectives

The characteristics of fusion protein-driven sarcoma offer several very promising therapeutic perspectives. First, the fusion proteins themselves are very interesting targets. Several lines of evidence suggest that the fusion proteins play a prominent role in the tumorigenesis of the corresponding sarcoma. Fusion-driven sarcomas usually have a low mutational burden, and often, the translocation is the only recurrent cancer-associated aberration [151,203]. Additional mutations, if present, are secondary to the translocation event and are only present in subsets of sarcoma cells [204]. Taken together, these findings indicate that the fusion proteins are the main drivers of tumorigenesis in these cancers. Furthermore, genetic silencing studies in a variety of entities have shown that tumor cells are addicted to the fusion protein activity and undergo cell death upon depletion [205,206,207]. These results suggest that fusion proteins are among the most promising therapeutic targets in translocation-associated sarcomas. The success stories with targeted therapies directed against PML::RARA and BCR::ABL in acute promyelocytic and chronic lymphocytic leukemia, respectively, but also the more recent clinical experience with inhibitors of kinase fusion proteins found in sarcoma, underscore this notion. Strikingly, only the kinase fusion proteins contain druggable domains that can be exploited for direct inhibition with small molecule drugs (Table 2).

In the vast majority of cases, however, no such domain is available, and for a long time, indirect interference with fusion protein activity has been considered the only valid option. Such an approach requires a deeper understanding of the biology of the individual fusion proteins and the proteins that act in concert with them [208]. However, proteolysis targeting chimera (PROTAC) molecules or molecular glues [209,210] could be game changers. These molecules bind to a target and induce its degradation without the need for direct inhibition, theoretically allowing the induction of specific elimination of potentially any protein.

A second therapeutic perspective arises from the close lineage association of some sarcomas. A prime example in this context is rhabdomyosarcoma, where the entire cellular hierarchy known from the normal muscle lineage is also present in the tumor, starting from stem cells to proliferating progenitors to committed and differentiated cells [211]. An important function of the fusion protein is to block the differentiation process and maintain (the majority of) the cells in a proliferative state. For this, the fusion proteins may keep the molecular machinery regulating differentiation in an anti-differentiation state. Manipulation of involved pathways downstream of the fusion protein may trigger differentiation of tumor cells. In the case of PAX3::FOXO1-driven rhabdomyosarcoma, inhibition of the RAS-ERK pathway leads to myogenic differentiation, similarly to normal muscle precursor cells [211,212]. These findings suggest that induction of terminal differentiation could be a useful therapeutic approach for sarcoma, in which the fusion protein acts as a gatekeeper of differentiation.

Finally, an interesting therapeutic opportunity may arise from the expression of neo-genes. Some of these genes produce spliced and polyadenylated transcripts that are ultimately translated. Interestingly, while such neo-genes were first associated with EWSR1 fusion proteins, when examining 18 other oncogenic chimeric transcription factor-driven sarcoma types, exquisitely specific neo-transcripts were identified for each of these entities [130]. From a cancer immunotherapy perspective, these neo-genes may offer an intriguing approach if their translated neo-peptides can be recognized exquisitely as tumor-specific antigens.

## 8. Conclusions

At first glance, the fusion oncogenes that drive sarcoma tumorigenesis are a very heterogeneous group of proteins. This suggests that a therapeutic interference with the activity of these proteins must be individualized for each sarcoma entity. However, a closer analysis of their mechanism of action reveals a somewhat different picture. Different fusion proteins can be assigned to groups with similar mechanisms of action. For example, the primary mechanism of action of several fusion transcription factors appears to be the recruitment of HAT activity to lineage-specific, regulatory elements in the genome. A small number of fusion partners act as HAT recruiters in various cases, such as members of the NCOA and FOXO families. Furthermore, the EWSR1 gene is involved in gene fusions in at least 11 sarcoma entities, suggesting that the relevant functionality of the EWSR1 protein can cooperate with different fusion partners and in different cellular contexts. Finally, several epigenetic fusion proteins act by opposing the activity of the Polycomb repressor complex, resulting in reactivation of gene expression.

Hence, these similarities in the mechanism of action might be exploited for the development of therapies that work in different sarcoma entities. In any case, they argue for a more molecular-based classification of sarcomas.

## Figures and Tables

**Table 1:** Markers for immunohistochemical detection of fusion proteins.

Fusion Protein	Sarcoma Entity	Antibody Target	References
PAX3::FOXO1	Alveolar Rhabdomyosarcoma	Breakpoint	[8]
SS18::SSX	Synovial Sarcoma	Breakpoint	[9,10,11,12]
NAB2::STAT6	Solitary Fibrous Tumor	STAT6	[13,14,15]
WWTR1::CAMTA1	Epithelioid Hemangioendothelioma	CAMTA1	[16,17]
FUS::DDIT3	Myxoid Liposarcoma	DDIT3	[18,19]
ALK-fusions	Inflammatory Myofibroblastic Tumor	ALK	[20,21]

**Table 2:** Potential therapeutic drugs for sarcoma with kinase fusion proteins.

Kinase	Sarcoma Entity	Potential Therapeutic Drugs
ALK	Inflammatory myofibroblastic tumor, Leiomyosarcoma	Crizotinib, Ceritinib, Alectinib, Brigatinib, Lorlatinib
ROS1	Inflammatory myofibroblastic tumor	Crizotinib, Entrectinib
NTRK	Inflammatory myofibroblastic tumor, Congenital fibrosarcoma, Adult fibrosarcoma, Dermato- fibrosarcoma protuberans	Larotrectinib, Entrectinib
BRAF	Spindle cell sarcoma, Myxoinflammatory fibroblastic sarcoma, Myxofibrosarcoma	Dabrafenib, Vemurafenib
